# Treatment of early resistant schizophrenia: a case report

**DOI:** 10.1192/j.eurpsy.2023.1567

**Published:** 2023-07-19

**Authors:** S. Benhammou, M. Raissouni, H. Kisra

**Affiliations:** 1 Hospital Arrazi of sale, rabat, Morocco

## Abstract

**Introduction:**

Early-onset schizophrenia begins before the age of 18. Drug treatments are mainly based on antipsychotics, preferably atypical antipsychotics, which have fewer side effects compared to first generation antipsychotics.

Resistant schizophrenia is defined as an inadequate response to two different antipsychotic treatments for a sufficient duration and dosage. Clozapine is the only drug treatment currently approved for patients with resistant schizophrenia, for which the risk of aganulocytosis must be monitored.

In order to derive a clear benefit, different scientific organizations, recommend the use of Clozapine as early as possible, and they state that there is little evidence to support the use of very high doses of antipsychotics.

**Objectives:**

We seek to determine the effectiveness of clozapine treatment in the management of early schizophrenia resistant to more than two antipsychotic treatments.

**Methods:**

Description of a case of early resistant schizophrenia , in a 16-year-old girl, put on clozapine in comparison with the data of the literature.

Discussion: through articles published on google scholar, pubmed, and science direct

**Results:**

Treatment with clozapine, showed efficacy in the case of early schizophrenia resistant to several lines of antipsychotics, including disappearance of auditory and visual hallucinations and delusions.

**Conclusions:**

The efficacy of clozapine treatment in early resistant schizophrenia raises the question of its use in first line from the beginning of the schizophrenic disease, however its side effects and its difficulty of follow-up raise questions in relation to its use.

**Disclosure of Interest:**

None Declared

